# Medical Topography, Climate and Yellow Fever of Rio De Janeiro, Brazil*1. Relatorio sobre medidas de salubridade reclamadas pela cidade do Rio de Janeiro, e acerca da Febre Amarella em particular, para subir a Augusta Presenca de S. M. O. Imperador, pelo Dr. Francisco de Paula Candido, Medico de sua Magestade o Imperador—premeiro secretario da Camera dos deputados—lente da escola de Medicina—Presidente da commissâo sanitaria, da junta central de Hygiene, e da Academia Imperial de Medicina, &c. Rio de Janeiro, no Typographical Nacional. 1854. Folio, pp. 51.Report upon the sanitary measures required in the city of Rio de Janeiro, and upon Yellow Fever in particular, to be submitted to the Emperor. By Dr Francisco de Paula Candido, &c. &c. Folio, pp. 51, with maps and tables. May, 1854.1*a*. The author presented a report, bearing very nearly the same title, in May, 1853, printed in 8vo., forming a neat pamphlet of 50 pages, to which occasional reference may be made.2. Observaçōes ácerca da Epidemia de Febre Amarilla do Anno de 1850, no Rio de Janeiro, colhidas nos hospitaes e na poly-clinica pelo Dr. Roberto Lallemant, (native of Lubeck,) Cavalleiro da Ordem Imperial de Christo do Brazil; Commendador da Ordem Imperial de S. Estanistáo da Russia, Medico da Enfermaria dos Estrangeiros da Santa casa da Miseracordia, e durante a epidemia da Enfermaria dos Estrangeiros dos lazaretos da Ilha do Bom Jesus e de N. Sra. do Livramento: Medico do Hospicio de Pedro II; Membro correspondente de Sociedades Medicas da Suecia, Prussia e Saxonia: 8vo. pp. 152. Rio de Janeiro, 1851.Observations on the epidemic Yellow Fever of the year 1850 in Rio de Janeiro, collected in Hospitals and general practice. By Dr. Robert Lallemant, (of Lubeck,) &c., &c.3. Descripçāo da epidemia de febre amarella que grasson na provincia do Cearáem 1851 e 1852 pelo Doutor Liberato de Castro Carreira. Formado pela Escola de Medicina do Rio de Janeiro, socio effectivo da Academia Medica Homœopathica do Brazil, &c., &c. 8vo. pp. 91. Rio de Janeiro, 1853.Description of Epidemic Yellow Fever, which prevailed in the province of Ceará, (Brazil,) in 1851 and 1852. By Liberato de Castro Carreira, &c., &c.

**Published:** 1855-11

**Authors:** W. S. W. Ruschenberger

**Affiliations:** U. S. Navy


### MEDICAL EXAMINER.

NEW SERIES.—NO. CXXXI.—NOVEMBER, 1855.

---

### ORIGINAL COMMUNICATIONS.

---

*Medical Topography, Climate and Yellow Fever of Rio de Janeiro, Brazil.** By W. S. W. Ruschenberger, M. D., U. S. Navy.

The city of St. Sebastian, or, as commonly called, the city of Rio de Janeiro, dates its foundation from about the middle of the sixteenth century. It is now the largest, as well as the oldest, city in America, south of the equator. In December, 1854, its population was estimated to be not less than 300,000; a census in 1850, returned 285,000; for this and other reasons, it is believed the estimate does not exceed the truth.

This city, the capital of Brazil, is situated in latitude 22° 55′ south, and longitude 43° 09′ west from Greenwich, on the western shore of an extensive bay, the extreme diameters of which are about twenty miles, more or less. The sheet of water is pear-shaped, and broken in many places by islands of considera-

*1. Relatorio sobre medidas de salubridade redamadas pela cidade do Rio de Janeiro, e acerca da Febre Amarella em particular, para subir a Augusta Presença de S. M. O. Imperador, pelo Dr. Francisco de Paula Candido, Medico de sua Magestade o Imperador—premeiro secretario da Camera dos deputados—lente da escola de Medicina—Presidente da commissâo sanitaria, da junta central de ble size; its shores are moulded into coves and bays, points and headlands. The back ground is formed of precipitous mountains, varying in height from 1500 to 3000 feet, which constitute a most picturesque scenery, clothed in the luxuriant vegetation of the tropics. Its geographical position makes it a commercial centre for trade with India, Oceanica, South America and Europe; ships of almost every nation are found, in the course of a year, lading or unlading cargo at this port.

The site of the city is a marshy plain, studded by lofty hills of granite, or granitic gneiss. The soil on the hill sides is remarkably thin, and, in any climate of less moisture and temperature would be inadequate to support the vegetable growth upon it. In the valleys it is deeper, an alluvium being washed from the declivities by the rains, and deposited upon a thick bed of

Hygiene, e da Academia Imperial de Medicina, &c. Rio de Janeiro, no Typographical Nacional. 1854. Folio, pp. 51.

Report upon the sanitary measures required in the city of Rio de Janeiro, and upon Yellow Fever in particular, to be submitted to the Emperor. By Dr Francisco de Paula Candido, &c. &c. Folio, pp. 51, with maps and tables. May, 1854.

1*a*. The author presented a report, bearing very nearly the same title, in May, 1853, printed in 8vo., forming a neat pamphlet of 50 pages, to which occasional reference may be made.

2. Observações ácerca da Epidemia de Febre Amarilla do Anno de 1850, no Rio de Janeiro, colhidas nos hospitaes e na poly-clinica pelo Dr. Roberto Lallemant, (native of Lubeck,) Cavalleiro da Ordem Imperial de Christo do Brazil; Commendador da Ordem Imperial de S. Estanistáo da Russia, Medico da Enfermaria dos Estrangeiros da Santa casa da Miseracordia, e durante a epidemia da Enfermaria dos Estrangeiros dos lazaretos da Ilha do Bom Jesus e de N. Sra. do Livramento: Medico do Hospicio de Pedro II; Membro correspondente de Sociedades Medicas da Suecia, Prussia e Saxonia: 8vo. pp. 152. Rio de Janeiro, 1851.

Observations on the epidemic Yellow Fever of the year 1850 in Rio de Janeiro, collected in Hospitals and general practice. By Dr. Robert Lallemant, (of Lubeck,) &c., &c.

3. Descripção da epidemia de febre amarella que grasson na provincia do Cearáem 1851 e 1852 pelo Doutor Liberato de Castro Carreira. Formado pela Escola de Medicina do Rio de Janeiro, socio effectivo da Academia Medica Homœopathica do Brazil, &c., &c. 8vo. pp. 91. Rio de Janeiro, 1853.

Description of Epidemic Yellow Fever, which prevailed in the province of Ceará, (Brazil,) in 1851 and 1852. By Liberato de Castro Carreira, &c., &c.

clay, which underlies nearly the whole of the district, and keeps the surface of the earth from becoming dry by natural drainage.*

When the city was commenced, on the Punta Calabouça, the vicinity was almost constantly overflowed by pools of stagnant water, which were supposed to be prejudicial to the general health of the population. Staunton, Macartney and other voyagers notice this condition, which remained up to the time of Pedro I., when the marsh was partially drained and improved. A look from the convent of San Bento, or a ride towards the imperial palace of San Christovâo, will satisfy the observant traveller of the real nature of the topography, and lead him to believe there are sources enough of miasm in the neighborhood to render the place unhealthy. Intermittent fevers prevail at all seasons along the shores, and upon the islands of the bay, especially amongst those of the population who are imperfectly nourished and sheltered; occasionally it is severe and fatal in its effects. In 1845, there was considerable mortality and suffering on the Ilha do Governador, which was attributed to extensive marshes by Doctor Joao José Vieira, who was appointed by the government to investigate the subject.†

Placed immediately beneath the tropic of Capricorn, Rio de Janeiro is under all the climatic influences common to equatorial and intertropical regions.

The granite hills around the city, as well as the expanded surface of the bay, constitute reflectors of the calorific rays which augment the temperature and humidity, which are not abated much by the winds because they are interrupted by the disposition of the mountains. This circumstance would render Rio de Janeiro less habitable were it not for the evergreen vegetation, which in turf and woods, covers the plains and hills, and the abundance of water which falls in cascades from almost every elevated point: these concur to temper the ardor of the climate. Nevertheless, the atmosphere is more or less impregnated with vapors, and sometimes covered with clouds, which hang about the mountain peaks, and cause, on some days, a suffocating heat, called by Brazilians *mormaço*—a sultry time—which is so debilita-

*Travels in the interior of Brazil, principally through the northern Provinces, and the gold and diamond districts, during the years 1836-41. By George Gardiner, M. D., F. L. S., &c. Second edition. London, 1849.

†Annuario Politico, p. 46.

ting as to deprive one of the aptitude for intellectual or muscular exercise, as well as the powers of digestion, and lead to a belief that the temperature is much higher than indicated by the thermometer. Meteorological observations made during thirty years, by Bento Sanches Dorta, and published in the "Memorias da Academia Real das Sciencias de Lisboa," show that the annual mean temperature of Rio de Janeiro was 73°.04 F., nearly 10° more than the mean temperature of Lisbon, although this city (Rio) is 16 degrees nearer to the equator. Consequently the heat of Rio de Janeiro, which is so much felt, particularly on cloudy days, is chiefly due to the nervous excitability and debility of the organism, caused by atmospheric humidity, and not to the excess of the thermometric scales, which is even below that of other localities in the same latitude.

The observations of A. M. de Mello, hourly made at the Observatory, show an increase of 2°.11 F. over those of his predecessor, Dorta. This augmentation of the annual mean temperature is attributed to the clearing of the forests in the interior and in the vicinity of the city, which not attracting rain as frequently as formerly, the atmosphere and earth are not washed as often by summer showers; besides, the land wind, not receiving exhalations from the multiplicity of leaves, the air is not so much freshened as when the forests were abundant, and, for this reason, it is supposed, the temperature has been greater; being now 75.151° F., and not 73.04° F., as it was in the past century.*

It is a fact generally attested by those well acquainted with Rio de Janeiro, that rain is not now as frequent as formerly. It is a vulgar tradition, that fifty years ago, when friends separated in the morning, they took leave of each other *until after the thunder shower of the afternoon*, which was an invariable phenomenon in the summer seasons particularly, but now this regularity of the afternoon rain does not exist. According to the observations of Dorta, at the close of last century, the annual mean fall of rain was 55 inches, and the number of rainy days was 140. According to the observations of A. M. de Mello,

*Relatorio sobre a Salubridade da Cicade de Rio de Janeiro e a Febre Amerella em particular, Para subir a Augusta Presença de S. M. O. Imperador por su reverente e respeitose medico Dr. Francisco de Paula Candido, Presidente da Junta da Hygiene Publica. Rio de Janeiro, 1853.

only 46 inches of rain fell in the year 1851, and the number of rainy days was 103, yet the year was not considered a dry one.*

Storms accompanied by electric discharges, so common formerly in the summer season, are now much less frequent. In the first year of Dorta's observations, there were 77 thunder storms over the city and its vicinity; and, according to Mello, there were only 18 in the year 1851. It is proper to state that in conversation, Colonel Mello expressed an opinion that observations had not been made for a period sufficiently long to establish this supposed change in the electrical constitution of the atmosphere. Dr. Roberto Lallemant, in his Observations upon the Yellow Fever of 1850, says, that for four or five years, thunder showers had sensibly diminished, and in the hot seasons of 1849–1850, they almost totally disappeared.†

The magnetic tension is, according to Baron Eckwege, 21 vertical oscillations per minute; and the inclination 28° 47′ 33″, south.*

The atmospheric pressure is subject to the variations observed in tropical countries; the least pressure corresponds to the humid and cloudy period of summer, namely, December, January and February; and the greatest to the dry days of winter, namely, May, June, July and August.

The monthly mean temperatures, according to Farenheit's scale, and the monthly mean pressure of the atmosphere, stated in millemetres, reduced to the temperature of 32° F., are exhibited in the following table, which has been prepared from tables politely presented to me by Colonel Antonio Manoel de Mello, Director of the Imperial Observatory at Rio de Janeiro.

*Climatologia em geral: em particular, o clima do Rio de Janeiro. Por Joaquim José de Medeiros. There para o doutorado em Medicina. Rio de Janeiro, 1852.

†Observaçōes acerea da Epidemia de Febre Amerella do Anno de 1850 no Rio de Janeiro, colhidas nos hospitaes e na polyclinica pelo Dr. Roberto Lallemant, (Natural de Luebeck.) Cavalleiro da Ordem Imperial de Christo do Brazil; Commendador da Ordem Imperial de S. Estanistáo da Russia; Medico da Enfermaria dos Estrangeiros da Santa Casa da Misericordia, e durante a epidemia da Enfermaria dos Estrangeiros dos lazeretos da Ilha do Bom Jesus e de N. Sra. do Livramento; Medico do Hospicio de Pedro II; Membro correspondente de Sociedades Medicas da Suecia, Prussia e Saxonia. 8vo. pp. 152. Rio de Janeiro, 1851.

*Monthly mean temperature and pressure of the atmosphere at Rio de Janeiro, for* 1851, 1852, 1853, *arranged from the observations made at the Imperial Observatory, by* A. M. de Mello.

☞The temperatures are according to Farenheit's scale. The height of the barometer is stated in millemetres, reduced to the zero of centigrade, or 32° F.

| | Mean Monthly Temperature. | | | Barometric Observations. | | |
|---|---|---|---|---|---|---|
| | 1851 | 1852 | 1853 | 1851 | 1852 | 1853 |
| | | | | mm. | mm. | mm. |
| January - | 84.824 | 79.316 | 80.474 | 754.388 | 755.330 | 755.059 |
| February - - | 81.649 | 80.402 | 80.416 | 754.779 | 755.911 | 754.677 |
| March - - - | 79.293 | 80.945 | 77.802 | 755.621 | 756.456 | 756.911 |
| April - - - | 79.550 | 76.875 | 79.187 | 755·699 | 756.714 | 756.216 |
| May - - - | 73.104 | 71.932 | 72.775 | 758.910 | 760.579 | 758.219 |
| June - - - | 69.805 | 71.980 | 70.734 | 760.106 | 761.128 | 761.395 |
| July - - - | 71.061 | 70.894 | 71.212 | 759.571 | 760.477 | 761.354 |
| August - - - | 70.474 | 70.028 | 72.035 | 759.210 | 760.015 | 758.667 |
| September - - | 68.122 | 71.760 | 74.139 | 759.382 | 759.305 | 757.617 |
| October - - | 73.545 | 72.129 | 73.792 | 756.178 | 757.758 | 756.773 |
| November - - | 74.915 | 77.217 | 77.932 | 753.260 | 754.980 | 755.361 |
| December - - | 76.676 | 81.786 | 77.257 | 755.095 | 754.511 | 755.084 |
| Mean of the year. - | 75.171 | 75.583 | 75.680 | 756.843 | 757.768 | 757.277 |

A luxuriant vegetation, an abundance of water, and the action of heat upon a locality excluded from a free circulation by the surrounding mountains, assist in augmenting the humidity which is noticed in the atmosphere of Rio de Janeiro. The observations of Dr. Friere Allemao, demonstrate that the humidity varies between a minimum of 42, and a maximum of 100 degrees of Saussure's hygrometer, the mean being 85. M. Pissis, in his work, presented to the Academy of Sciences of Paris, proves that the humidity of Rio de Janeiro is double that of Paris.

The absolute humidity increases in the course of the year, from July to the following February, when it reaches its maximum; it then decreases in the same ratio until it falls to the least quantity. In the course of the day, the same march is observed: the maximum is in the early morning; it increases until 5 o'clock, P. M., and decreases with the cooling of the earth; this course is more regular in summer than in winter, when the humidity is nearly the same throughout the twenty-four hours. These hygrometric details, reported by M. Pissis, are confirmed by the observations of Colonel Mello, made with the condensing hygrometer of Regnault.

*Hygrometric condition of the Atmosphere at Rio de Janeiro, for the years* 1851, '52 *and* '53, *arranged from observations made at the Imperial Observatory by the Director,* A. M. de Mello.

| | CONDENSING HYGROMETER OF REGNAULT. | | | | | | | | |
|---|---|---|---|---|---|---|---|---|---|
| | Monthly Mean for the year 1851. | | | Monthly Mean for the year 1852. | | | Monthly Mean for the year 1853. | | |
| | Dew Point, or Temp. of Condensation. | Tension of Vapor. | Weight of water in cubic metre of air. | Dew Point, or Temp. of Condensation. | Tension of Vapor. | Weight of water in cubic metre of air.* | Dew Point, or Temp. of Condensation. | Tension of Vapor. | Weight of water in cubic metre of air.* |
| | Deg. Fahr. | M.M. | Grammes.* | Deg. Fahr. | M.M. | Grammes. | Deg. Fahr. | M.M. | Grammes. |
| January . . . . | 70.647 | 18.470 | 18.160 | 72.330 | 19.500 | 19.600 | 72.660 | 19.720 | 19.760 |
| February . . . | 69.617 | 19.210 | 19.000 | 73.297 | 20.070 | 20.200 | 74.674 | 20.070 | 20.970 |
| March . . . . | 70.912 | 18.590 | 18.100 | 74.428 | 20.940 | 20.800 | 72.264 | 19.500 | 19.540 |
| April . . . . . | 70.275 | 18.250 | 18.100 | 69.887 | 18.020 | 18.100 | 73.603 | 20.310 | 20.420 |
| May . . . . . | 62.740 | 14.120 | 14.500 | 65.370 | 15.440 | 15.800 | 67.620 | 16.660 | 17.080 |
| June . . . . . | 61.147 | 13.340 | 13.700 | 64.275 | 14.480 | 15.300 | 63.725 | 14.660 | 15.270 |
| July . . . . . | 60.111 | 12.900 | 13.100 | 64.088 | 14.770 | 15.100 | 63.176 | 14.300 | 14.830 |
| August . . . . | 64.162 | 13.690 | 14.100 | 64.504 | 15.020 | 15.400 | 65.262 | 15.390 | 15.750 |
| September . . . | 61.507 | 13.510 | 14.100 | 66.340 | 15.970 | 16.200 | 67.926 | 16.870 | 17.100 |
| October . . . . | 64.407 | 14.930 | 15.300 | 66.302 | 15.970 | 16.200 | 65.085 | 15.290 | 15.660 |
| November . . . | 68.775 | 17.290 | 17.100 | 70.631 | 18.470 | 18.600 | 68.288 | 17.080 | 17.300 |
| December . . . | 69.385 | 17.600 | 17.600 | 73.693 | 20.440 | 20.400 | 69.814 | 17.910 | 18.100 |
| Mean of the year, | 66.124 | 15.830 | 16.200 | 68.750 | 17.290 | 17.600 | 68.684 | 17.290 | 17.500 |

* A gramme is equal to 18.43 grains, (apothecaries' weight,) or 15.4340 Troy grains. A metre is equal to 39.371 inches; a cubic metre is equal to 1072.8628 cubic feet.

Rio de Janeiro is not visited by winds of extraordinary force, such as are known in intertropical regions. A north east wind, (the land breeze) prevails from mid-night to mid-day; and a south east, (sea breeze) from mid-day to mid-night, there being an interval of calm generally preceding the commencemont of the sea-breeze. During the summer months, the sea-breeze begins later and ends earlier; and in the winter, it begins before mid-day and continues beyond mid-night. An east wind sometimes blows from daylight, and increases in force during the early part of the day. Winds from the northwest and southwest veer into the more regular winds; the first is frequently the precursor of thunder storms, which ordinarily accompany the second.

There are two seasons in Rio de Janiero, the summer or rainy season, and the winter or dry season; nevertheless, careful observation shows a meteorological disturbance and an irregularity of the weather about the period of the equinoxes, which, though not as decided as in temperate regions, bears a remarkable similarity to them. It is not uncommon to see sharp lightning, principally in the months of March, April, September and October, on the brightest tropical day with a clear atmosphere and blue sky, succeeded by a cooling of the air and change of wind; these phenomena, which characterize the changeableness of weather of temperate countries, are less perceptible here.

Alluvia of more or less recent clay, humus and sand form the soil upon which the city stands; the smaller hills are composed almost exclusively of clay, with here and there an exception of one which has a nucleus of granite, and form the artificial earth on which a greater part of the city is built. The humus forms a superficial bed in the lowest situations, and the sand borders the shores, and beneath a mixture of humus and clay constitutes the deeper beds which rest probably on granite. Different salts and many other substances would be found at greater or less depths, in certain places, if we may judge from the waters of the wells, some of which yield carbonic and hydrosulphuric acids.

Rio de Janeiro has in the environs eight springs of ferruginous waters, and the neighboring mountains supply an abundance of potable water, through two principal aqueducts, that of Carioca and of Maracana, which is poured forth at every corner through brass hydrants to supply domestic wants, and for the extinguishing of fire.

The ancient bed of the sea, upon which the city now stands, having been transformed into a marsh by deposits from the ocean and transportation of alluvium, was afterwards converted into a plane, by earth excavated from the neighboring hills. The city, thus levelled and built, according to the taste and circumstances of individuals, without regard to what public utility required, exhibited at a later period numerous defects. Scarcely any shed or declivity for the drainage of water,—a thousand yards from the shore, Campo de Santa Anna is only 5½ feet above the level of the sea—the narrowness of the streets, bad paving, the slightly permeable nature of the soil, give the city a dirty aspect, whenever a slight rain wets the soil ; waters deposited by the carelessness of the people, are retained and neglected by the police, until carried into the air by evaporation, to be respired for a long time, unless removed by the wind ; all these inconveniences attended the growth of the city from its earliest days, and now cannot be remedied without great difficulty.

The climate of Rio de Janeiro cannot enjoy the reputation of being very salubrious on account of its tropical position and the nature of its locality, but it is not, however, entitled to the mortiferous character attributed to it. In the year 1851, when the population was stated at 266,466 souls, of which 152,965 were males, and 113,501 females, the mortality reached 8,713, or three per cent., which is not excessive considering the ravages of the epidemic yellow fever. With the exception of yellow fever, which, according to some, appeared for the first time in 1849, and according to others, was sensibly felt in remoter periods, Rio de Janeiro has not been attacked by any of the great epidemics which often desolate intertropical countries. Paludal fevers, of the intermittent or remittent form, sometimes complicated with ataxic or adynamic phenomena, cause the greatest part of the mortality, principally after the evaporation of the waters of the summer, ordinarily in the months of February, March and April. Next to paludal fevers come diseases attributable to suppressed perspiration, especially in July, August and September, which is due to sudden changes of temperature and humidity, manifested by inflammations of the skin and chest. The alternate action of these two morbid causes, the intermittent element and the sudden changes of temperature and humidity, is probably productive of pulmonary phthisis which in all medical statistics stands at the head of the list.

A comparison of the present sanitary condition with that of more remote periods shows an increasing frequency in the pernicious character of intermittent fevers; and some practitioners affirm that yellow fever is an endemic modified by the condition of the atmosphere.*

Dr. Candido asserts that the dwellings of Rio de Janeiro, in a majority of instances, are better adapted to Lapland or Greenland than to the tropic region. They are erected in rows, often upon a level below that of the streets and gardens. Their dimensions are small. The roofs of tile, and low; often a wooden floor rests upon a damp soil, and constantly in a state of decay. They are badly lighted and illy ventilated.

In the houses of the opulent are spacious saloons which are reserved for the reception of guests, while the wealthy inhabitant sleeps in a close alcove, covered by mosquito nets or bars. The walls are covered with paper which retains the humidity of the air.†

According to Dr. Candido the atmosphere of Rio de Janeiro has become modified with the increase of its population and with the changes which civilization has wrought upon the habits of the people. Certain diseases inherent to the city, such as erysipelas, intermittent fevers, hyperhæmias, &c., have diminished, while phthisis, diarrhœa, scarlatina, typhoid and yellow fevers have increased in frequency and intensity. This change in the public health must be due, he says, to climate or other influence which society or civilization has brought with it.

Certain causes at all times, especially in our day, have attracted the attention of physicians and of governments, because observation has shown their influence, and science has explained their mode of action in destroying public health and in abbreviating life.

Air, water, locality and food, have been considered from the

* Joaquin José de Medeisos. Climatologia em geral, em particular, o clima do Rio de Janeiro, 1852.

† Relatorio sobre medidas de salubridade reclamadas pela cidade do Rio de Janeiro e acorea da febre amarella em particular, para subir à Augusta Presenca de S. M. O. Imperador, Pelo Dr. Francisco Paulo Candido, medico de sua magestade o imperador—primeiro secretario da camara dos Deputados, &c. Folio, Rio de Janeiro, 1854.

time of Hippocrates as the principal receptacles or vehicles of agents destructive to public health.

Whether the atmosphere may be deteriorated by a crowd of healthy or sick persons, by effluvia arising from organic decomposition, vegetable or animal, or by the quantity of aqueous vapor, is of little import: an air impregnated by organic emanations or moisture is always prejudicial to man.

The atmosphere containing *miasms*, (organic bodies in a *state of transformation*), acts, when absorbed, by their chemical properties as poison, or communicates their state of vibration, or movement of decomposition, to the principles of the organism.

The importance of the function of respiration, through the means of which air contributes to the maintenance of life, is manifest in all living beings: plants and animals of every kind, great and small, derive a part of their aliment from the atmosphere.

A few remarks will show the importance of the air or respiration to human life, and its influence in the production of diseases. 1. It contributes considerably to alimentation. 2. It may become the vehicle or means of conveying poisonous matter into the interior of the organism.

It has been demonstrated that a man breathes 260 cubic centimetres (13 cubic inches) of air at each respiration: he breathes 260 × 18 = 4,680 cubic centimetres (234 cubic inches) in a minute: he respires 4,686 × 60 = 280,800 cubic centimetres (14,040 cubic inches) in an hour; and 280,800 × 24 = 6,739 litres, (quarts) 200 cubic centimetres (336,960 cubic inches) in a day.

Of these 6,739 litres of air breathed in 24 hours, 6 per cent. represents the volume of oxygen, which, condensed in the blood, passes to the interior of the organism; that is, 404 litres, 34 cubic centimetres of oxygen, which is equivalent to 5,822.5 grs., or 1 pound, 2 ounces and 6 drams.

A pound of a substance introduced daily into the organism cannot fail to produce a marked influence upon the equilibrium of life.

It is perhaps in this way, chiefly, says Dr. Candido, that the miasmatic air of Rio de Janeiro has developed pulmonary phthisis, which diminishes in the country, and disappears in the wild forests; among the tribes of the *Coroados*, who are nomadic or errant, among the *Puris* and the *Naknenuks*, who inhabit the dense forests of Parahyba, of Pomba and of Rio Doce, no instance of pulmonary phthisis has been observed.

In the second place, miasms are generally denser than the air, (and therefore occupy an inferior position and never rise but a short distance into the atmosphere), assuming that they have the same density as oxygen, admitting, too, that the atmosphere contains miasms in the same minute proportion as it holds carbonic acid gas, that is $\frac{6}{10,000}$, the weight of miasms conveyed into the organism by the respiration of 6,739 litres of air in 24 hours. According to these premises, here admitted, the volume of miasms would be 6,739 litres $\times \frac{6}{10,000} = 40,434$ litres, whose weight is $40,434 \times 1.11 = 4.5$ grammes $=$ 1 dram and 8 grains. One dram and eight grains of putrid substances, such as miasms are, is a quantity, according to observations frequently repeated, more than sufficient to produce diarrhœas, typhus, algid and putrid fevers, plague, yellow fever, cholera, &c. Miasmatic absorption by the lungs alone is here calculated in its minimum, without taking into account absorption by the whole surface of the skin, or that effected through the solvent qualities of the saliva, and through the food and water we consume.

An atmosphere impregnated with miasms in the proportion of only six ten thousandths of its volume, is enough to render it pernicious and fatal to man.

Humidity is indispensable to those putrid emanations which alter the healthy condition of the air. The presence of excessive moisture in the air, by embarrassing cutaneous and pulmonary exhalation causes secretions to be returned back into the organism, where, acting as poisons to the organs they disturb the harmony of their functions and lead to the generation of morbid products; and, serving as a vehicle, provoke the evolution and diffusion of miasms in the atmosphere. But humidity by itself does not exert the same influence.

Under ordinary circumstances a man exhales from the skin and from the lungs eighteen ounces of water in 24 hours, or $37\frac{1}{2}$ ounces according to Dalton. These exhalations are imbued with, and thus set free from the organism, those organic matters which had become superfluous and foreign to it. When man is in a humid atmosphere, exhalation from his body diminishes, or even ceases if the surrounding *hygrometric tension* is at its *maximum ;* then, these superfluous or effete organic matters are retained in the blood, now become foreign substances to it, act as if they had been absorbed, and analogous disturbance occurs, if other organs, as the kidneys, intestines, liver or glandular system do not become supplementary to exhalation.

The vague expression, *influence of climate*, so constantly used may be rendered in most cases, by *presence of emanations* and by the *hygrometric* state of the atmosphere, which *emanations* and *humidities* depend upon topographical circumstances, the nature of the soil, relative position to the sun, prevailing winds, weight of the air, or accidental circumstances.

It is not solely the absolute quantity of aqueous vapor suspended in the atmosphere which determines its hygrometric state or *humidity*, but without the *tension* of these vapors, this tension depending upon the quantity of vapors suspended, and upon the *temperature* and on atmospheric pressure; circumstances which vary every hour, every day and every month. Wherever these or any one of these circumstances vary, on a great scale in the vast extent of the atmosphere, or on a small scale in the limited space of a city, or in a dwelling, the hygrometric state becomes modified in its respective extent. The air becomes more humid wherever evaporation is augmented, where the temperature is reduced, where pressure is increased, (supposing the air to be saturated). Where the circulation of the air is embarrassed, in a city, or in the interior of a dwelling, it becomes humid, because the accumulated vapors reach the maximum of *tension*, and are condensed into humidity ; in a city or in a house where the temperature is lowered, the accumulated vapors reach the maximum of tension and are condensed in rain or in humidity ; and the same result follows by augmenting the pressure or weight of the atmosphere.

Dr. Candido maintains that, (after dilgent study of the most enlightened authors on the subject, and direct experience), epidemics cannot exist without miasms or their equivalents ; that miasms absorbed either poison us by their chemical properties, or communicate their state of decomposition to the substance of our organs, particularly to the blood ; that the same miasms which pre-existing in a population, produce yellow fever under the influence of a certain *excitant*, would produce cholera, plague, or typhus, &c., under the action of *other* excitants respectively; that, in fine, without the intervention of other excitants than chemical affinities set in motion by local circumstances, emanations from any place can be *spontaneously developed* into the miasms of some of these scourges.

According to these premises—some of them directly demonstrated by eminent observers, especially by English physicians, other corollaries consequent upon the advances of organic chemistry, none of which have been contradicted, but always corroborated by facts here observed—we may set up a theory, that the true and radical means of preventing yellow fever, cholera, plague, and all pestilential epidemics, consists in preventing the formation of miasms and of humidity, and in promoting other conditions which maintain public health; because, it is miasms, humidity, water, improper food, insalubrious dwellings, bad habits, &c., which accumulate in the organism those elements which form the combustible of epidemics.

Dr. Candido repeats the assertion that the considerations upon which we must base our preservative measures against epidemics are, 1st, without miasms and humidity, or their equivalents, epidemics cannot exist; 2d, that the presence of pestiferous individuals and objects have sometimes been an *excitant*, the incendiary torch of epidemics, in places *where miasms or their equivalents* pre-existed. In support of these positions he gives some examples:—

As to the first—pre-existence of miasms and humidity—Sierra Leon, Gibraltar, Barcelona, Havana, Demerara, Barbadoes, New Orleans, when treating of yellow fever; the banks of the Ganges, the coasts of Coromandel, of Malabar, and the banks of the Indus, in the history of cholera; the delta of the Nile, Syria, Constantinople, Marseilles, when we speak of plague, offering as they do a vast field for investigation, confirm the cause of these epidemics; and exclude from their causes the circumstances of variety of races, habits, religion, food, latitude, longitude, &c., while the constant coefficients of epidemics—miasms and humidity—exist in those places. Cholera pauses before great elevations; the plague does not pass the cataracts of the Nile; yellow fever does not leave the coast; in Gibraltar it did not pass the Neutral

Ground; in the West Indies, slight elevations served to limit its progress; in Rio de Janeiro, a height of 400 feet and a distance of two leagues, were its insurmountable barriers, &c.; now, all these limits observed by the cholera, the plague, and yellow fever, may be regarded as the line of diminution and disappearance of miasms and humidity or their equivalents.

As to the 2d—An excitant or ferment developing epidemics.—The Island of Ascension, of Boa Vista, Barcelona itself, lighted up by the Bann, the Eclair, and by the Grand Turc, which, visiting those points, caused the appearance of yellow fever; on the coast of Brazil, Bahia, Pernambuco, Rio de Janeiro, Cearà, from St. Catherine's to Parà, at the two extremities of the empire, a distance of 25 degrees, it may be said, were consecutively affected by yellow fever, the outbreak being coincident with the arrival in those cities, respectively, of the slave ship Brazil from New Orleans, or rather Havana; of the Alcion, from Bahia, after that place was affected, of the barque Navarre from New Orleans, and many others from Pernambuco, Bahia, *Africa*, when those places were suffering from yellow fever,—the Margaret Hopping, the national vessel Carioca, proceeding from Rio, and Pernambuco already afflicted, all arriving in time to have ascribed to them the paternity of the yellow fever, constitute irrefutable testimony that these vessels carried an *excitant* which developed the fever in those places: the unprejudiced mind refuses to admit that so many coincident facts could be without any relation between the arrival of the vessels and the explosions of fever in places and under circumstances so different; and to regard them as merely accidental, on the extended coast of Brazil, without the least relation to the epidemic, is, in the opinion of Dr. Candido, a gross absurdity—it is to attempt to subject facts to a fixed idea —to influences merely local.

In the Lying-in Hospital at Vienna, it was observed that of 3000 women delivered, 500 were annually affected with epidemic *puerperal typhus*,—a frightful proportion of one in six. The intelligent physician of the establishment soon recognised that the pestilential *excitant*, which provoked the epidemic, was brought by the students, who, frequenting the anatomical theatres, came with their clothes saturated with putrid exhalations; it was remarked that the service and contact of the students who came from contending with adynamic fevers, in other wards and amphitheatres, was especially fatal to the parturients. Measures were taken to exclude these ambulant foci of infection from the maternity wards, and the epidemic disappeared.

If the exhalations with which the clothes of the students were saturated exercised an influence in another manner than as an *excitant*, which set in motion the decomposable matter—equivalent to miasms—which chemistry has shown to exist in the blood of parturient females; if the action was other than an excitant or ferment, if it was simply a poison, the bearers of these exhalations, the students, should have been the first to be poisoned; which did not happen, because they were not *parturient females*, that is, their blood did not contain fermentable matter, or the equivalent of miasms.

In the opinion of Dr. Candido, the causes of all epidemics of cholera, plague, yellow fever and typhus which have devastated parts of the world, may be stated in the following two propositions:—

"1. An atmosphere vitiated by miasms and humidity; occasionally or very often other causes, different in appearance, but which produce in the organism analogous or identical effects to those which proceed from miasms, always appear as constant co-efficients of epidemics, as the *materia prima* of these inflictions.

"2. An *excitant*, a *ferment* or pestilential focus, brought from a short or a great distance, provoking devastating epidemics from miasms, or products of other causes, but *equivalent* to miasms accumulated in the organism, is an incontestable fact, and which places in harmony the contradictory observations which constitute the chaos in the history of pestilential epidemics."

*What were the causes of yellow fever in Rio Janeiro?*

For a century prior to the year 1850, yellow fever had not appeared in Brazil, at least not as an epidemic.

Dr. Candido, in his report dated May 1853, says that heat is not a direct cause of yellow fever; that it is not most common in the *isothermic zone* (80°.6 F. to 86° F.), that is, in the equator of heat: the mean temperature of Sierra Leon is 86° F. Places where yellow fever most prevails, New Orleans with a mean temperature of 67°.72 F., Havana 77° F., are inferior in temperature to Parà, (where it is 81.5° F.) and other localities in which the disease is more benign. In Rio de Janeiro it was not, during the highest temperature, 79.34° F. to 81.5° F. in the months of January and February, that its prevalence was greatest. In those months the mean (the maximum of the year) was less in 1852 than in 1851, and as the whole epidemic was incomparably less intense in 1851 than in 1852, the epidemic intensity was in an inverse ratio to temperature.

The action of heat in the production of yellow fever is secondary:—

1. By promoting the decomposition of organic substances and the evolution of miasms.

2. By facilitating life in myriads of insects and other animals, as well as in plants, all of which become material for organic decomposition.

3. When an individual is saturated with miasms, by respiring a pestilential atmosphere, *insolation* will act directly as a determining cause which agitates and disorders, setting up fermentation in the liquids of the economy, which closes in black vomit; yet, heat is neither an exclusive nor an essential cause of yellow fever.

*Humidity.*—The hygrometric column, the general mortality, and the mortality from yellow fever fluctuate together, and are affected by the same *co-efficient*, as may be seen by reference to tables. There can be no doubt about the influence of humidity as here demonstrated; it renders recourse to other arguments and foreign labors unnecessary.

On the approach of autumn, from February to March, while the earth, still heated by the summer's sun, exhales vapors and miasms, these are condensed by the action of the cold air which flows from the south, humidity is then very great, as shown in the meteorological tables (above given): the effect increases in proportion to the augmentation of the cause; that is, the general mortality and that from yellow fever grow with increased humidity. The influence of humidity is incontestable; it is demonstrated by the observations made in the city of Rio de Janeiro.

*Atmospheric pressure.*—A diminution of the weight of the atmosphere, (which takes place in the same months in which meteorological observations exhibit an increased humidity, and which does not coincide with the maximum temperature), is a powerful cause of augmentation of humidity and miasms whose exhalation is always in inverse proportion to atmospheric pressure; in this respect meteorological observations are in remarkable harmony with the causes of yellow fever and other diseases which afflict the capital. It is clearly seen that a diminution of atmospheric pressure coincides with an increase of the epidemic and the mortality.

Meteorological observations teaches us that *humidity* accompanies *pari passu* the two mortalities; that diminution of atmospheric pressure (which facilitates the evolution of miasms) follows the same law; that a maximum temperature is not the most prejudicial to public health, and that a minimum is the most salutary for Rio de Janeiro.

One must shut his eyes to these facts to deny the influence which *temperature*, the *hygrometric state*, and *atmospheric pressure*, exercise on public health, and particularly on the production of yellow fever.

In his report, dated May, 1854, Dr. Candido says:—"I find in my notes, that in 1836, two American sailors were admitted into the Infirmary under my charge, in the Santa Casa da Misericordia, who were *yellow with great prostration* after delirium, &c. One died on the third day, but *post mortem* examination revealed no lesion except a serosity, which was notably yellow, in the pericardium, in the abnormal cavity and in the cerebral ventricles; I have no doubt now that those cases were yellow fever.

Dr. Lallemant says that scarcely had yellow fever appeared in his clinic when his colleagues declared, "This is not yellow fever; the disease does not reach Rio de Janeiro!" "But now that its existence in the city cannot be denied, each one asks, 'How is it possible that this disease should have penetrated our capital?'" But Dr. Lallemant, on the contrary, asks, "How is it possible that yellow fever should not have been developed in Rio de Janeiro?"

"My friend and colleague Dr. Valladão tells me," continues Dr. Candido, "that, some years ago, he observed a patient, a Prussian he thinks, whom he suspected was affected with yellow fever, but now he would denominate it *hæmorrhagic fever*.

"In 1823 an English sloop of war, the Bann, proceeding from Sierra Leon in March, infected the island of Ascension; in like manner another vessel of war, the Driver, arriving from Africa with her crew in perfect health, was infected at the island of Ascension after communication with the Bann; both vessels sailed for Bahia, but in spite of their visit the epidemic did not spread in that city.

"Some years preceding the present date, both prior to and after the discovery of gold in California, vessels and passengers from the United States, New Orleans, &c., from Havana, from Sierra Leon and other places on the coast of Africa, were received without precaution or fear of yellow fever, even when it prevailed in the ports from which they cleared.

"Upon the whole, in spite of the doubtful cases, in spite of the well ascertained case of the Bann and of the frequent reception of persons arriving from foci of yellow fever, the disease did not become epidemic in any part of Brazil.

"Pure contagion, then, was not the exclusive cause of the epidemic in 1849—1850; some other cause was applied in those climateric years which rendered the *febrile element* active.

"The most recent and important researches relative to epidemics and pestilential diseases, and the light of organic chemistry, show that these scourges are the natural consequence of causes extrinsic to the organism—meteorological, miasmatic or others—which disturb the physiological functions, whether they act in the organism, or where they encounter in it the materials or *intrinsic* causes which under the influence of *extrinsic* causes develope fatal products. Let us examine these *extrinsic* and *intrinsic* causes, and their mode of action at Rio de Janeiro in the year 1850."

*Causes extrinsic to the organism.* For many years prior to 1850, there were no regular or consecutive meteorological observations; but it is a common remark among the inhabitants of Rio de Janeiro that the regular winds (land and sea breeze), the thunder-showers, almost invariable in summer, the clearness of the atmosphere after the long prevalence of the winter winds from south and southwest, have been very much modified for some years.* During the last months of 1849 and the com-

* Dr. Lallemant states that probably as a consequence of this meteorological change, an epidemic occurred about 1846. Every body fell sick without apparent cause, but as scarcely one (at any rate very few) died, both the profession and the public gave it little consideration. It was called the Polka Fever, because it was as common as the monomania for the polka dance.

The patients presented themselves with headache, the conjunctivæ injected, and general pains, principally in the loins, white furred tongue and slight pains in the hypochondria; in some cases, irritation of the stomach with bilious vomitings, &c. The disease prevailed so extensively among seamen, that business among the shipping in port was almost entirely suspended. The same fever appeared again in January, 1848, and Dr. Lallemant states that he then suggested to a friend, that under favorable circumstances for incubation, the disease might become yellow fever.

mencement of 1850, on the eve of the epidemic, the sun appeared red, of the color of blood, and might be gazed at with impunity; and the moon wore the same aspect. The burning winds from the north, almost to the exclusion of the sea breeze from the southeast, prevailed during this period, and the impression is that for some years this breeze had become feeble. Meteorological observations of subsequent years show that the explosion and culmination of the epidemic occurred in the months of the greatest humidity, and consequently, when the atmosphere contained the greatest quantity of miasms and emanations. Mortality increased in proportion to the increase of humidity and diminution of atmospheric pressure.

These phenomena show that anteriorly to, upon the eve of, and during the epidemic, great quantities of aqueous vapor and carboniferous emanations were suspended in the atmosphere of the city. Now, these vapors, emanations and gases being suspended in the atmosphere, porous bodies enveloped in it were necessarily impregnated, that is, they absorbed these gases and emanations; they entered into the structure of the wood employed in buildings, floors, partitions, movables, furniture, &c., and certainly penetrated the organism of man, through whose lungs passed daily 16 pounds of this atmosphere. Every thing submerged in the same medium must have been alike impregnated and *saturated* with these vapors, emanations or gases.

To these vaporous masses, which, when suspended in the atmosphere, refract the luminous rays, are added emanations from organic substances of many kinds, such as are found in the filth and offal of a city, in stagnant pools, &c.

Joined to these carboniferous vapors, and like them absorbed by various structures and by the pores of the body, these organic emanations accumulate within us, and kept by porous bodies, remain in reserve until called forth by a humid air, when they escape transformed or developed into pestilential miasms.

This retention or preservation of gases and miasms is undeniable. Coal absorbs 90 times its volume of ammoniacal gas; old wood, *humus*, organic matters in like manner, when dry, absorb gases and exhale them when exposed to humidity; spontaneous combustion of coal is due to the enormous quantity of hydrogen which charcoal retains when absorbed; a putrid or ammoniacal odor is exhaled from old houses when closed, from dirty places, from depots of timber, from the earth itself, when the air becomes damp. Other examples might be adduced to show the absorption of gases by porous substances, and their exhalation by the pressure of humidity.

Nor can the infection of the organism by air be denied. Daily 1,739 litres of air, equal to more than 16 pounds, pass through the human lungs. Now this air being impregnated with organic emanations in the proportion of one part in a thousand, it carries into the organism, where a greater part remains, six drams (360 grains) of organic emanations. Such is a diagram of the impregnation of an organism living in an atmosphere contaminated by miasms in the proportion of one part in a thousand.

No one doubts that the atmosphere, habitations and all organized beings were impregnated by gaseous exhalations or miasms when the epidemic of 1850 broke out.

In the height of the epidemic, in March, 1850, the atmosphere was washed by copious and long continued rains, and seemingly appeared to be purified when the skies were clear for a short time. It was supposed that the scourge would abate on this account; but on the contrary, the epidemic increased with the rains. "Noticing, then, the increase of disease when the air ought to have become pure, the inference was that the cause of the epidemic was not, at least not exclusively, in contact with the atmosph re ; the contagionists and infectionists were, in the opinion of Dr. Candido, wrong. The smells and ammoniacal odor which became sensible in the dwellings of misery and generally in the afflicted districts, the sickening of a great number of individuals in the same house, as from accidental circumstances, when the heavy rains made those places damp, &c., led to the belief that the rain, which washed the atmosphere externally, rendered the air within dwellings damp, and that the action of this humidity was to attract and set at liberty the miasms, previously absorbed by various materials in the houses, and in this manner, the inhabitants who breathed the air in them, became infected."

With this humidity, by its action, another species or equivalent of miasms appeared as a substitute where there was none, and where there was, to combine in the production of fever. *A diminution of cutaneous perspiration and pulmonary exhalation,*

which, restraining the exit of matters now become foreign in the economy, determined their retention in the torrent of the circulation, where their influence was the same as if an equal quantity had been respired. Collard de Martigny has shown by analysis that the air *expired* contains $\frac{3}{1000}$ of putrescent organic matters, which are constituted of *albumenoide;* other analogous observations go to establish beyond a doubt that a suppression of these two functions, or very great abatement in their activity, infects the economy exactly in the same manner as a miasmatic atmosphere does. The quantity of these febrile materials retained in the system by a cessation of perspiration, may be estimated, if we bear in mind that a man, under ordinary circumstances, exhales eighteen ounces of aqueous vapor, charged with these *albumenoid* principles, in twenty-four hours. When pulmonary and cutaneous transpiration cease, if the kidneys (which act often as in reserve to relieve the economy of supplementary secretions), do not promptly give exit to those products which have become foreign, and which should have passed off by the skin and lungs, the system will retain them, and would be infected just as if miasms had been directly breathed.

With these incontrovertible facts in view, it is easy to understand that, the organism being saturated with miasms, either directly by infection, or indirectly by the retention of foreign matters, the individual who respires for some time a miasmatic atmosphere (which *excites* decomposition) carries with him, in his system, the *excitant:* as soon as cutaneous and pulmonary exhalations cease or diminish, and the supplemental or vicarious secretions of the kidneys fail to give exit to the miasms breathed or to their equivalents retained, and to the products of decomposition arising now from the action of the *excitant* on the materials of the economy, zymotic disorder or fever will necessarily appear. It is for this reason that persons who left the infected atmosphere of the city of Rio de Janeiro, or coming to it to be impregnated with miasms in 1850, fell victims to the fever in Tijuca, in Petropolis and other places, beyond the focus of the epidemic, which was not propagated in those points, because the *general excitant for its diffusion*, a miasmatic atmosphere, was wanting.

It was observed in 1850, that the unfortunate who, saturated with the infected air in the focus of the epidemic, changed to a cooler place, as the neighboring mountains, or a more humid and miasmatic situation as to old, closed-up houses, thus disturbing the equilibrium established between the infection of the system and the depurating exhalations in a high temperature, almost infallibly became victims of yellow fever. In damp, badly-ventilated houses; in the filthy little rooms of sailor boarding-houses, near the bay; in all places in which emanations or miasms were absorbed and preserved in porous substances, it broke forth with increased energy in damp weather.

In general, new ships, and especially those on their first voyage, were least affected; old ships, particularly those whose cargoes consisted of putrescent organic, or porous substances, were most gravely affected. These facts show the influence which miasms exercised in the production of yellow fever, and agree with observations by others on porous mineral coals.

Between the 1st of April, 1853, and 31st of March, 1854, one hundred and thirty-five vessels loaded with coal, anchored in this port; of these 76 suffered from yellow fever and 59 escaped. Of the 59 which did not suffer, a third only, more or less, anchored at the health anchorage. Of the 76 which suffered, nearly one-half anchored near the quays.

The angle of the bay which constitutes the *health-anchorage* is a receptacle of the filth which flows there from the neighboring shores; the visiting steamer which boarded vessels anchored there very often made her way through sea-weed and filth: on board of her it was often noticed, in consequence of the smell, that the air made stagnant in this corner by the neighboring declivities, was converted into a true *hygrometer*. From the position of the headlands humidity always prevails in this place.

The natural inference is that, more than in any other anchorage, the coal here emitted its miasms under the action of humidity, and that, invigorated by the miasms of this region, those which were emitted by the coal became productive of yellow fever, a property which the miasms from the coal do not in themselves possess, and they do not produce yellow fever in other cities where these same ships have been.

It results from these complex observations; 1st, that old miasms, or those retained long in porous substances or recently closed places, pass through the phases of decomposition which communicates to them different properties, and augments generally their deleterious qualities; 2d, and that miasms are always the primary matter, the indispensable condition of the epidemic.

Let us now examine the question, if yellow fever is caused by miasms, as miasms have long existed in this city, how is it that yellow fever has not existed ever since the existence of miasms in Rio de Janeiro and other places? This is a favorite objection of those who will not take the trouble to visit and examine the places most affected, the holds of ships, sailor boarding-houses, &c., where, it is observed, that fever is always in relation to the miasms. This question does not belong exclusively to Rio de Janeiro; it, as well as its solution, applies to all countries visited by epidemic yellow fever, and constitutes a cardinal point in the history of this scourge.

Those who have discussed this question (contagionists and infectionists) have fallen into an error by assuming that all miasms are alike. From this mistake arose the theory of *concentration* under which Tomassini was content to account for the yellow fever of Leghorn, in 1804, as by an infectionist.

Now, miasms are not always the same, whether evolved from different animals or vegetables, or from the same animals and vegetables. Many circumstances cause these products of organic decomposition to vary; the principles which cause them to vary are the *excitants* of decomposition and the meteorological conditions, besides the diversity of substances from which they proceed. For example:

Air contained in an enclosure where there is wood in state of eremacausis, or decay, yields *carbonic acid gas* exclusively; if a sufficient quantity of hydrogen gas be added to this air water is found exclusively, but not an atom of carbonic acid. This was demonstrated by De Saussure.

Liebig affirms that animal matters in a state of decomposition yielded different products, according to meteorological conditions; they give ammonia in cold climates, and nitric acid within the tropics.

The emanations which produce the aromas, from the oil of the seeds of Sambucus nigra, oil of turpentine, oil of lemon, different emanations from each one, as also in general, the emanations which produce the aroma of flowers, result from the oxidation which these oils undergo. Geiger has demonstrated in like manner that the peculiar smell of musk results from its decomposition or putrefaction, that is, from emanations which its putrescent decomposition produces. The products of all these decompositions are therefore different; no one regards them as identical.

Liebig states that a solution of cyanogen, before reaching its ultimate and definite transformation, passes successively through eight changes or different products.

The emanations from marshes are evidently different from those which arise from a graveyard: analysis confirms what the evidence of the senses announces.

The juice of the grape fermenting, that is, decomposing slowly, yields wine which consists of alcohol, carbonic acid, ethers, &c., but later, by placing it under another condition, all the alcohol is converted into vinegar. The products of decomposition vary with the phases of this decomposition.

No one can confound the smell nor the pathological effects of drying rose leaves, and the decomposition of thousands of plants, with the smell and pathological effects of a dissecting-room or of a grave.

The matters whence they emanate, the circumstances which influence all these exhalations and gases, are different; how is it possible to assert that such emanations are, in all cases, the same, more or less concentrated?

In respect to these organic compositions the lessons of analysis are of the greatest importance; because they show that a most insignificant circumstance will entirely vary an organic product: for example, the very same elements arranged in a line, in a circle, or in a triangle, may be sufficient to form of them different compositions, innocent, agreeable, or poisonous, or at any rate three different substances constituted of the same elements: thus;

| | | |
|---|---|---|
| Essence of Cinnamon. | Carbon | 79.52 |
| | Hydrogen | 6.40 |
| | Oxygen | 14.06 |
| Essence of Bitter Almonds. | Carbon | 79.66 |
| | Hydrogen | 5.56 |
| | Oxygen | 14.88 |
| Essence of Prunus padus. | Carbon | 79.35 |
| | Hydrogen | 5.68 |
| | Oxygen | 14.09 |

are composed of the same elements, and very nearly in the same proportions, but they are very different in their chemical, physical and therapeutic properties. Sugar, gum and cotton are in a like manner composed of the same elements, nearly in the same proportions; but no one makes clothes of gum, nor pastes paper with sugar, nor sweetens his coffee with cotton.

Dr. Candido, to show the difference of miasms emanating from different substances, made the following experiments:

1. By means of a mixture of ice and salt he condensed, on the surface of a glass sphere the emanations from the hall of the school of medicine when filled with students. The liquid obtained gave the characteristic odor of the *human halitus.* This liquid was introduced into Volta's eudiometer which filled 1-10th of its capacity; the eudiometer being closed, gently warmed, the remaining 9-10ths of its capacity (filled with air) were saturated by emanations from the liquid, which was manifest by rapidly opening and closing the superior opening of the instrument: for 24 hours, and even for two days, the same *smell of halitus* remained. He withdrew the condensed liquid and left in the instrument only the air saturated with emanations; the smell of halitus remained unchanged. Carefully avoiding the loss of air, he introduced into the eudiometer 1-10th of its volume of lime water, then exposed the instrument to the direct rays of the sun, and afterwards concentrated them by means of a lens; then submerging the eudiometer in water and opening the lower valve, the liquid did not rise, showing there had been no condensation of oxygen into water nor into carbonic acid; by opening the upper valve, the smell of human halitus was still perceived. Recent pulmonary exhalations are not easily decomposed, not even under the action of the sun's rays and of lime in contact with air.

2d. Afterwards, but at the same period, the eudiometer was emptied of distilled water in an atmosphere super-saturated with the foul, putrid emanations from a sewer; the eudiometer filled with this air gave the odor of the filthy sewer which infects the city. Lime water equal to 1-10th of its capacity was passed into the eudiometer, and the instrument exposed for two minutes to the rays of the sun, and gently agitated: the lime water became turbid. Being submerged and the lower valve opened, the water rose nearly 1-12th of the capacity of the eudiometer, and moreover, it was found, on opening the superior valve the putrid odor had entirely disappeared. If it be contended that the rise of the water in the glass was due exclusively to carbonic acid which must have contained the putrid exhalations, the combustion of the miasms which disappeared must be admitted; then the disagreeable, putrid odor disappeared *instantaneously* and *completely;* a glass tube moistened with chlorohydric acid was surrounded by white vapors when plunged into these exhalations; the formation or pre-existence of ammonia is thus established. Without lime water, and employing only sun-light concentrated by a strong lens, the result was the same at the end of four minutes. Putrid emanations from ordure are changed into carbonic acid, ammonia, and water when exposed to the sun's rays in contact with the air particularly when lime is present.

These emanations or miasms are different then from those exhaled from the lungs of animals.

The diversity of physiological emanations even from the human species is so noticeable, so characteristic, says Dr. Candido, that whoever passes through Brazilian forests may recognize by the smell alone, that is by emanations, the places of sojourn of indigenous tribes, and these physiological exhalations persist many days; though there are no porous dwellings nor clothes to retain them, they attach themselves to the humus, to trees, to dry leaves, &c. Who cannot recognize the odor of the slave ship, even for weeks after her cargo has been disembarked?

It being established that the nature of miasms is varied by many different causes, it is not wonderful that, not having, in previous epochs, evolved the miasms proper to produce yellow-fever, others were happily evolved which produced other diseases; this epidemic, (yellow fever,) would not appear, except when the meteorology, or the excitants of the formation of miasms, caused to appear those which are productive of the disease.

Different excitants, which are only meteorological conditions, cause the products of decomposition or miasms to vary, and also to give origin to different pestilential diseases—these *excitants* very often do not contribute in a single material atom in the formations of miasms; they sometimes act by their pressure alone, communicating their state of vibration; at other times they enter into combination with substances, and determine their decomposition.

In the experiment of De Saussure, the rotten wood caused the formation of water, by communicating its state of vibration to the oxygen and hydrogen which, without this vibration do not combine at the ordinary temperature: but the rotten wood contributes nothing to the formation of water; it serves only as an *excitant.*

The ferment of bread or of beer put in a solution of cane sugar communicates its vibration to the elements of the sugar, and causes them to be converted into alcohol and carbonic acid; but not an atom of the ferment enters into the products developed in the sugar.

The same solution of cane sugar, which forms alcohol and carbonic acid under the action of ferment, when boiled with a few drops of sulphuric acid is converted into *glucose* or grape sugar.

The same cane sugar by dry distillation is converted into carbonic acid, carburetted hydrogen, carbonic oxide, empyreumatic oil, into vinegar, &c.

In contact with pus, sugar is converted into lactic, butyric acids, &c.

On the other hand rags, or old fabrics of linen, cotton and wool boiled with nitric acid, yield grape sugar or glucose.

Starch mixed with saliva is converted, in a minute, into grape sugar or glucose; the sweet taste which follows the chewing of bread or sea-biscuit in a short time is owing to the saccharine transformation of the starch contained in wheat by the saliva.

But old rags, starch, &c., burned or distilled, that is, subjected to other excitants, yield products which are very different from grape sugar.

Hence, Dr. Cardido infers that *different excitants* produce in organic substances different products. The first products of organic decomposition vary according to the organic substances decomposed, and according to the *excitants* of this decomposition; so that it may happen, on account of this very variety, that the same product may *accidentally* result from different substances: how, from that which is not seen, nor felt, nor susceptible of experimental demonstration, can we draw a conclusion that miasms are always the same, varying only in degree of concentration !

But aside from experimental demonstration, who would be bold enough to maintain that the *excitant* of small-pox, measles, yellow fever, hooping cough, puerperal typhus, &c., maladies which are propagated by emanations ; who will assert, that the cause of all these diseases is the same, because they all arise from organic decomposition ! It would be better to confound, says Dr. Candido, the aroma of the rose with that of the onion, or that of coffee with tar.

The decomposition of organic bodies, called *eremacausis*, *fermentation* and *putrefaction*, by which, after being deprived of life, they are separated into gaseous products, is due to a loss of the equilibrium in which their molecules were held, and this loss of equilibrium may be occasioned, either by meteorological conditions (heat, humidity, electricity, &c.,) or by the intervention of another body, whose state of molecular vibration is communicated, and, by its presence, destroys this equilibrium : or by the chemical affinities of the elements which constitute their immediate principles : the equilibrium once destroyed and the molecules set in disorder, they then freely obey their chemical affinities and form products which vary for each body. Let us not deceive ourselves, for, in living bodies, it is not the chemical affinities of the different immediate principles nor the vital forces, which ordinarily, without great meteorological influences, spread disorder in the living economy, disturbing the equilibrium in the composition of its fluids and solids : it is almost always, at least in great epidemics, the movement of *vibration*, which, through the means of miasms or other equivalent, is carried to the interior of the organism by respiration, by cutaneous absorption, by diet, &c. The sausages or smoked meats of Wurtemburg, referred to by Liebig, afford irrefragable proof of this *vibratory* action.

This assertion will seem strange to him only who is not aware of the energy with which fermentation and putrefaction proceed in deoxidation and other chemical phenomena by a mere vibration communicated to the principles which are fermenting.

The fact of an excitant provoking decompositions in the organism, is confirmed by many observations recorded in the annals of medicine. Take the following examples:

1. A wound from the point of a scalpel used in dissecting a decomposing corpse excites a malignant fever, phlegmons, and suppurations, in *organs* often very distant from the point of injury.

2. Blood, cerebral matter, bile, &c., when *corrupt*, produce vomiting, algid fever, typhus and death when applied to the skin deprived of its epidermis, or to a wound. (Majendie.)

3. The smoked meats of Wurtemburg sometimes produce emaciation and fatal decline without the least inflammatory indication. (Liebig.)

4. A small portion of putrid animal matter injected into the veins of a sentenced criminal, has produced typhus and death.

5. A solution of sugar and ferment injected into the veins of an animal enters into fermentation, which is propagated through the vascular system, and produces *typhoid* fever. (Claude Bernard.)

6. Corrupt water consumed on board of ship is often a cause of erysipelas, diarrhœa, typhus, &c.

7. In Saulier, (France,) in the year 1773, in the Mother Church of Santo Antonio, there accidentally met 120 children for their first communion, and other persons; depositing a heavy corpse in its resting place, a coffin which had been buried thirty-three days before, was broken, from which was diffused through the church a horribly fœtid stench. The 120 children, the curate, the grave digger and all sickened of a putrid fever with hæmorrhage (like the yellow fever), eruption, &c. (Walker.)

In all these cases, the cause (miasm) was the exciting medium of decomposition in the liquids or solids of the organism. In proportion as these decompositions are analogous to those which occur in fermentation and putrefaction, so are the products analogous: in fact, in these cases, as in pestilential epidemics, the inseparable companion of putrefaction, ammonia, appears in the sweat, in the saliva, in the urine, in the atmosphere respired by the sick, and in the form of sulphate in the evacuations. (Liebig).

Meteorological conditions may determine the formation of miasms productive of yellow fever from the filth and other sources of exhalations in a city.

But appropriate or peculiar excitants, as a ship laden with organic substances which have received the impulse of decomposition in the port of departure, impregnating the vessel and her crew with miasms, may also, undergoing this decomposition, convert into miasms of yellow fever the pre-existing miasms and those arising from foci of filth in the presence of the excitant. But the *excitant* coming from without will be inoperative if it does not meet with miasms or foci of miasms, and besides this, the coincidence with certain meteorological conditions, heat, humidity chiefly, which favor their transforming action, a coincidence which, not always occurring, the invasion of epidemics is more rare, even where there are miasms.

Dr. Candido thinks he has demonstrated that there was in Rio de Janeiro a sufficient quantity of miasms to serve as the *materia prima* of the epidemic of 1850; but miasms having been here for a long time, and in analogous meteorological circumstances, which, doubtlessly, should have produced it in preceding years, without we conclude that the epidemic yellow fever should not appear without some *excitant*, *ferment*, or what they may please to call it, intervened in the climateric year of 1850, to transform these pre-existing miasms or their *equivalents*, into miasms productive of yellow fever.

The explosions of yellow fever in the capitals of the Provinces of Brazil, always coinciding with the arrival of vessels from places affected with it, is opposed to the principle laid down by Dr. Candido, and the corroborating facts, besides those observed here, are referred to in other parts of the world where, without preconceived notions, the determining cause of yellow fever has been investigated, though not referred to as proof of the principle he sustains, namely—that an excitant or ferment coming from without is the incendiary torch which often lights up the epidemic.

In fact, the fever appeared in Bahia, Pernambuco, Ceará, Pará, Rio de Janeiro and St. Catherines, only after the arrival, on their respective coasts, of the North American brig Brazil, employed in the slave trade; the French ship *Alcion* proceeding from Bahia already attacked; the Danish brig Pollux, and the

Brazillian ship Carioca, proceeding from affected ports; the American barque Navarre, the Brazillian war steamer Affonso, the Portuguese corvette D. Joao I., the English packet Petrel, all proceeding from Bahia while the fever prevailed, and the American brig Margaret Hopping, &c.

The American barque Hercules arrived in December, 1849, directly from Philadelphia; some of her sailors died of yellow fever in the Misericordia Hospital.

As in the cases of the English sloop of war Bann, in the island of Ascension, in 1823; the corvette Le Dauphin in Cadiz, in 1800; the ships Grand Turc and Taille Pierre, in Barcelona in 1821; the Swedish ship Dygden, in Gibraltar in 1828; the English steamer Eclair, at the island of Boa Vista in 1845, it was always observed in the different ports of Brazil, that the epidemic proceeded from a ship, or from a small number of houses previously affected, from which the fever was propagated in a few days.

In Bahia, the disease was propagated from the brig Brazil to the whole city. The Brazil was direct from New Orleans; she had cases of yellow fever on board, and arrived (December 1849) at the time or just before the explosion of the fever. In Pernambuco it began in the Alcion, and extended to the ships Constantine and Josephine, lying close to her, and thence to the English infirmary at Boa Vista, and Recife, and afterwards the fever spread through the city. At Pará it spread from two vessels above named. At St. Catherines the disease spread from the Margaret Hopping.

Many persons in Rio de Janeiro believed that the contagion of yellow fever had been imported from ports of the United States, New Orleans and Philadelphia. Dr. E. D. Fenner, of New Orleans, in his valuable report of the epidemics of Louisiana, Mississippi, &c., published in "The Transactions of the American Medical Association," vol. vii. 1854, says, page 449, "It was currently reported, during the summer [1853], that this fever was brought to us by vessels from Rio de Janeiro," a report shown to be unfounded. The testimony favors the idea that, both at Rio de Janeiro and New Orleans, the epidemic was of local origin.

From the work of Dr. Lallemant, already cited, we learn that on the 13th December 1849, the war steamer Dom Affonso arrived at Rio de Janeiro, announcing that yellow fever had broken out at Bahia, and that from thirty to forty sickened daily; that in Brazilians and acclimated foreigners, the disease was mild, but very fatal among the recently arrived.

The next day the Portuguese corvette Dom Joâo I., arrived from the same port, having on board 209 soldiers, besides her regular crew. Five persons had sickened on the passage, of whom two died. The corvette was put in quarantine.

On the 24th December, the British packet Petrel arrived from England, via Pernambuco and Bahia, with some sick on board, of whom two died at the entrance of the port.

But no one of these vessels nor patients exercised any influence, at least apparently, on the state of health of Rio de Janeiro. "The evil was latent among us before we received the first news of the existence of the epidemic at Bahia."

The American barque Navarre, Capt. Little, with a crew of nine men, left Bahia in the latter part of November, and after a passage of 12 days arrived at Rio de Janeiro on the 3d of December. As nothing had been then heard of the epidemic at Bahia, she was admitted to free pratique; the captain sold the vessel and the crew dispersed. Some of these men lodged in Franck's American Boarding house in the street of Misericordia.

"When I made my visit on the 28th December to the infirmary for strangers, in the Santa Casa da Misericordia, my attention was especially directed to two new patients, whose disease appeared to me peculiar. The most prominent symptoms were a yellow color of the conjunctiva and skin, vomiting of dark colored liquid, hiccough, suppression of urine; in one hæmorrhage from the mouth and anus, delirium," &c. These two patients were:—

1. Enquist, a lad from Finland, who had arrived direct fourteen days before in the Russian brig Wolga.

2. Anderson, a Swede, entered the Santa Casa under an order of the American Consul; sojourned in Franck's tavern.

The first patient died the following night, and the second forty hours afterwards. On the 30th December Dr. Lallemant pronounced these cases to be yellow fever, but his opinion was not concurred in. But on the 4th January, 1850, another case presented.

3. Alexander Wilson, with some suspicious symptoms. He belonged to the American barque Hercules, direct from Philadelphia, and boarded at Franck's tavern. On the 9th of January he left the hospital re-established.

4. Josiah Baker, an American sailor, was admitted on the 5th of January, with almost the same symptoms as patients 1 and 2. Baker and Anderson were both from the Navarre, and both lodged at Franck's. Baker died 40 hours after admission with the same symptoms as his unfortunate companion.

The Navarre arrived from Bahia on the 30th December ; the barque Hercules had discharged some of her men, for mutinous conduct, and three of them boarded at Franck's.

Dr. Lallemant immediately visited Franck's tavern, but found no one sick.

5. Mathew Donelson, (admitted January 7th,) American, from the Hercules ; like No. 3, he lived at Franck's. He died in 44 hours after admission.

Visiting Franck's tavern the same day, Dr. Lallemant found two patients.

6. Thomas Lamerton, an American, with the fever completely developed, and the spleen very much enlarged, consequent upon attacks of fever on the coast of Africa.

7. H. Marshall, an American, then in the commencement of the disease.

Both cases were instantly sent to the Santa Casa. Patient No. 6 died in 48 hours, and Marshall (No. 7) after 12 days treatment left the hospital cured.

8. January 8th, William Hamlin, an American sailor from Franck's tavern, admitted in the commencement of the fever. He left the hospital on the 12th, but returned eight days afterwards with copious black vomitings, and died at the end of 24 hours.

9. Meogy, an American sailor from the barque Hercules, and like No. 3 and 5, a boarder at Franck's tavern, where he sickened on the 8th of January. Declining to go to the hospital, he was treated at Franck's and was re established in five days.

Opposite to Franck's and in the immediate vicinity of it were two other sailor taverns ; one kept by Wood, an Englishman, and the other by Auguste Hourdé, a Frenchman; there were sailors living at both of them. All these sailors visited from tavern to tavern. On the 8th of January in the Santa Casa da Misericordia was—

10. Thomas Fox, an English sailor living at Wood's; with symptoms of the fever; he left the infirmary cured on the 12th.

11. Robert Luff, an Englishman, for many years a resident in Brazil, addicted to intoxication, lived at Wood's. He sickened on the 8th, entered the hospital on the 10th with the fever fully developed, and died in 48 hours.

These two cases induced Dr. Lallemant to visit Wood's tavern, where he found that

12. Wood himself,

13. The wife of Wood, and

14. Lenschau, his German clerk, had had the fever mildly; Lenschau was then convalescent.

15. Auguste Hourdé, master of the third tavern, entered the infirmary on the third of January with slight fever; on the 5th, feeling well, he asked his discharge, but on the 14th he returned with violent fever, and died on the 20th.

16. The wife of Hourdé sickened on the 17th with slight fever, and was re-established on the 21st of January.

17. A French sailor, name forgotten, sickened at Hourdé's at the same time, and recovered in a few days.

18. Washington Sands, a mulatto, an American sailor living at Wood's, sickened on the 10th of January, entered the hospital on the 12th with violent fever, grew better and left on the 18th with eyes very yellow.

19. Lawrence Lattrow, an American sailor, at Franck's tavern, where he was found, at 9 o'clock P. M., Jan. 13th, almost moribund, having been sick three days. He was immediately conveyed to the hospital, where he died in a few hours.

20. Joseph Patrick Rodgers, an American sailor, a boarder at Franck's, sickened with fever, and was admitted into the infirmary on the 17th, and died on the 20th of January.

Enquist, the subject of case No. 1, came directly from Finland to Rio de Janeiro, and lived on the declivity of the Castello, just behind Franck's house, at a height of 20 or 30 feet. Whenever he descended the hill to the beach of Santa Luzia, he necessarily passed Franck's house, where there was always one or more Swedes, as Anderson No. 2, for example, with whom Enquist entered the hospital; there the Finlander could find some one who spoke his language, and perhaps it was the only tavern in the city where the Swedish language was spoken. Therefore it is probable that Enquist had some communication with Franck's house.

In the latter days of his life, Enquist had been frequently on board of the Russo-finland vessels anchored in the harbor; indeed he sickened on board of one of them, whence he was conveyed to his lodgings. And eight days after his death, yellow fever suddenly broke out with violence on board of two of those Finland vessels, the Norna and Niord; almost at the same time, the captain, mate and a sailor died; and on the 10th of January several sailors from these vessels, and a Swede from the Swedish ship Scandia, were carried to the strangers' infirmary of the Santa Casa da Misericordia.

And further in the street Misericordia, between the houses of Wood and Hourdé, and opposite to Franck's, is the house of a German merchant, whose daughter, who was the wife of her father's partner, had returned a few weeks from Altona, in the ship Marie Christine; in her company was a German girl named Amalia Elizabeth Peersman. The family lived at the hotel Pharoux, but breakfasted at the house in Misericordia street. The servant sickened on the 8th of January, and died in Misericordia street on the 13th, with a violent attack of yellow fever.

From the begininning of January, the sailors of the Marie Christine daily visited the house in Misericordia street, opposite to Franck's, and suddenly several of that ship's company fell sick.

From on board of the Marie Christine, amidst the crew of which the germs of the disease existed, sailors went daily to the house of Christiano Hess, on the shore D. Manoel, to buy fresh meat, and suddenly his German cashier from Petropolis, who sold the meat to the sailors, was taken violently with the fever; but on the 14th of January, six days after the invasion of the disease, he was out of danger, and was re-established at the end of three weeks.

On the 8th of January, Dr. Lallemant reported the existence of yellow fever in Rio de Janeiro.

The government directed a meeting of the Imperial Academy of Medicine to be called. The diagnosis of Dr. Lallemant was questioned, chiefly on the ground that the cases had occurred almost exclusively in his own practice; a single case was reported by Dr. Feital, which came from Bahia in the steamer D. Pedro, and died on the 29th December in the Marine hospital. A committee was appointed, and reported in such a manner that doubts existed in the minds of some; but two days later, no member of the Imperial Academy could deny that yellow fever was present.

On the 17th of January, three patients from the Russian schooner Norna, four from the Swedish brig Alfhild, three from the Danish schooner Marie Christine, one from the Russian schooner Niord, all suffering from the same symptoms, entered Dr. Lallemant's infirmary. Along Misericordia and the neighboring streets, several cases had occurred, and at the next meeting of the Academy a member announced that every physician must be convinced of the existence of yellow fever in Rio de Janeiro—and such is a history of its commencement in the year 1850.

In the latter half of January, the cases became too numerous to be registered. The disease passed from house to house and from street to street. Whites suffered most, next the mixed races, and the pure blacks almost entirely escaped. But the great mortality was among unacclimated foreigners. The rich and the poor suffered alike. The fever prevailed most fearfully in March, April and May.

In two months Dr. Lallemant states that he received patients into his hospital from 95 vessels of different nations, so that almost all the languages of Europe were spoken at the same time under one roof.

Men suffered more than women, and those between 16 and 40 years of age suffered most.

It was estimated that there were in the year, 100,000 cases of fever, of which 10,000 died.

Numerous hospitals were established for the reception of yellow fever cases. Into these hospitals there were admitted up to 31st of May, 2,086 patients, of which 1,036 died. In the three following months 216 cases were admitted, of which 87 died.

A small steamer, the Santa Isabel, was employed (and is still)

to visit ships in the harbor, to convey the sick to the hospital. A flag displayed on the bowsprit signified a yellow fever case on board; the steamer, provided with medical officers and necessary appliances, immediately came alongside and conveyed the patient to the shore, where he was furnished with everything requisite, even to clothing, free of charge.

Dr. Lallemant says, that the hospitals established for the treatment of yellow fever, were like those of other countries, when the exigency of circumstances did not permit the construction of appropriate edifices: although not perfect, he feels obliged to declare, from what he saw and heard, that all strangers and foreign governments especially, have abundant reasons for being grateful to Brazil for having, in a calamity so terrible to foreigners, provided so considerately and extensively for this class of the sick.

Dr. Candido describes three periods or stages of yellow fever. The symptoms of the first, are loss of appetite, thirst, a pasty, bitter taste, the tongue at first moist and pale, afterwards with a white or yellow fur in the centre, with red edges and tip, yellowish or greenish vomitings of ingesta, pains in the epigastrium, and sometimes also in the belly; constipation; rarely icterose.

Difficulty of respiration; breath hot and of a peculiar odor; rarely epistaxis.

Pulse full and frequent; throbbing of the temporal and carotid arteries; intumescence of the jugular veins.

Cephalalgia, eyes brilliant, watery, very sensible to light, pupils dilated; insomnia, or comatose condition; difficulty of speaking; contusive pains in the limbs and back; tongue and lower lip tremulous. Urine red and scant. Decubitus dorsal; color of the breast, neck and face red; skin hot and dry; conjunctivæ injected.

Second period. Tongue furred, with papillæ of the same color, or normal, or dry and of a satiny red, with a darker stripe in the centre; more or less frequent vomitings of liquid ingesta, bilious, color of chocolate, black (like coffee-grounds or tar) or of blood; severe pains in the stomach, heat like the scalding of black vomit, (precursory); belly painful, sometimes tympanitic, generally flaccid; boborygmata, eructations; right hypochondrium sometimes sensitive; evacuations yellow, obscure, black, commonly fœtid; excoriations of the anus.

Respiration irregular and difficult; abundant epistaxis; blood does not readily coagulate.

Pulse frequent and soft, seldom full and hard; pulsations of the heart relatively stronger than those of the radial artery.

Slowness of reply, confusion of ideas, insomnia, delirium, comatose condition, sobbing, jactitation.

Urine turbid, diminution of quantity, and not without sediment. The ammoniacal transformation which rapidly supervenes prevents the deposition of extractive matters.

Conjunctiva yellow, a livid circle around leech bites, red color at some points, and yellow at others, the first disappearing on pressure to become yellow, but soon resuming the primitive color —scarlatinose spots with furfuraceous or scaly desquamation, furunculæ, petechiæ, sudamina.

Third period. Lips cracked, dry, teeth fuliginous, sanguineous exudations from the entire mucous lining of the mouth; tongue dry, cracked, red, presenting here and there small coagula of blood; severe pains in the stomach and belly, particularly in the iliac fossæ, the patient shrinking from the slightest touch; black vomitings increased in frequency; the evacuations, ordinarily of the same nature, become insupportably fœtid; rectorrhagia—in some cases diarrhœa; intense inflammation of the tonsils, causing asphyxia.

Dyspnœa; expired air cold and fœtid.

Pulse filiform, irregular, or soft and frequent.

Anxiety, sobbing, eyes dull, comatose condition, or restlessness, the patient desiring to throw himself out of bed, deafness, delirium, carphologia, or picking at the bed-clothes, subsultus tendinum, convulsions, in some cases of an epileptic form, complete prostration, indifference.

Entire suppression of urine, urethral hæmorrhage.

Hæmorrhage from all solutions of continuity, from the conjunctiva, afterwards gangrene, decubitus dorsal, with the extremities in supination, parotid abscesses, cold extremities, diminished temperature, cold sweat, gangrenous ulcerations over the trochanters and sacral region.

To these symptoms, noted from the observation of nearly 4000

cases in hospital, may be added others frequently met in private practice, which, in the opinion of Dr. Candido, are worthy of especial attention. He enumerates the following:

Defective memory; from the moment of invasion, or in one or two hours, the patient is unable to explain; he is conscious of loss of memory. Sometimes intense pains in the lower extremities, in the lumbar region, &c., constant pain in the supra-orbital region, at least in the first period of the disease; a weakness in convalescence, totally disproportioned to the severity or benignity of the disease: in some cases the intelligence remains clear till the moment of dissolution.

Secretion of mucosities from the mouth, œsophagus and internally so excessive that their expulsion is very difficult, and they require to be removed.

Incessant thirst.

The pulse throughout compressible or soft, for the sygmoid aortic valves being closed and the muscles which compress the arterial system being relaxed, the elasticity of this system is deprived of these auxiliaries, and yields to any pressure whether the artery, provided it be full, contains a greater or less amount of blood. Dr. Candido never failed to observe this compressibility or softness of pulse from the onset in serious cases.

Miliary eruption, urticaria, &c., even in individuals not attacked, but threatened.

The following is a summary of the post-mortem appearances observed in the persons who died of yellow fever in the marine hospital of Santa Isabel at Rio de Janeiro.

*Digestive apparatus.*—In some cadavers, inflammation of the œsophagus, its mucous membrane softened in patches, covered with a glutinous liquid, more or less dark colored, analogous to the dark liquid vomited during life. In most instances the stomach contained a black liquid; in a few, it was yellow or reddish; the mucous membrane was of a red color, sometimes so loaded as to simulate ulcerations, ecchymoses; it was softened; the pylorus presented more extensive excoriations than the cardia. The color, texture, and liquids found in the duodenum were of the same character as those of the stomach; the large intestines suffered the same lesions, but were not so frequently affected. In some instances the liver was normal in condition, in others it was enlarged and marked with red spots, or of a friable texture. The gall-bladder always contained bile, in variable quantity, dark colored, of a muddy green, or of normal color; change of its density was seldom observed.

*Urinary apparatus and peritoneum.*—The bladder contained more or less urine dense, dark, yellow; sometimes of a normal color—or it was contracted and without liquid. Its mucous membrane was more dense and injected about the neck than in the fundus. In most instances no change was noted in the kidneys; sometimes their volume was augmented and their color was darker than they commonly are. The peritoneum in a great number of cadavers was injected at points, and marked by lead-colored spots.

*Nervous apparatus.*—No alteration in the consistence of the cerebral mass worthy of notice was observed in some cases; but in some it was more flaccid than natural. The meninges and encephalon were injected; in the latter, the injection being in points more or less distinct: serous, slight sero sanguinolent effusions in the ventricles, and in the cavities of the arachnoid; effusion of blood in the cerebrum was rarely observed. Yellow or sanguinolent serosity was found in the interior of the rachis; the envelopes of the medulla were more or less engorged, particularly in the sacro-lumbar region.

*Respiratory apparatus.*—Passive congestions and partial engorgements in some part of the lungs with minute crepitation; signs of inflammation in the mucous membrane lining the bronchia; these changes were not constant.

*Circulatory apparatus.*—Effusion, in small quantity, of yellow or sanguinolent serosity both in the pericardium and endocardium, without a trace of inflammation in either organ. In many cases dark blood with or without soft coagula was found in the cavities of the heart and in the great vessels: in some cases they were empty.

*External appearances.*—The color of the skin was citrine yellow with violet-colored spots, entirely violet, or in some cases lead-colored: in some, petechiæ and sudaminæ, principally on the breast and belly; a livid circle around leech-bites, and sites of vesicatories; in very few the skin was marked by furfuraceous desquamation; conjectivæ in two cases were so much injected as to simulate large coagula: ulcerations over the sacrum and great trochanters. In general all solutions of continuity presented a gangrenous aspect, and on the surface as well as through nearly all the tissues an infiltration of a yellow liquid was observed.

*Treatment.*—"If," says Dr. Candido, "yellow fever results from decomposition in organic principles, and this decomposition is set up through the intervention of an excitant, as I have endeavored to show, the object of treatment is then,

"1st. To destroy or at least neutralise the action of the excitant, and to modify the process of decomposition, which is in general attained by the agents which neutralise the excitants.

"2d. To eliminate from the economy all the *materia prima* of these decompositions, whether from a part already under the process, or another part not yet under the decomposing influence.

"To attain these two ends it is necessary:

"To remove the patients from an infected atmosphere, to one rich in *ozone* and oxygen, but not into a very different temperature.

"To throw into the torrent of the circulation by friction, drinks, and by all possible means, those agents which posssess the property of modifying, retarding or arresting the process of decomposition, such as oxygen (inhaled), chlorine, creosote, camphor, aromatics, &c.

"To accelerate all the secretions from the moment of invasion, particularly the intestinal secretions, and those of the skin, kidneys and salivary glands—even to force, artificially, copious secretions by vesicatories; the effects of a large blister on the epigastrium when the symptom of a burning sensation there threatens black vomit, are really admirable.

"To maintain the regular exercise of all the functions, stimulating those which require to be accelerated, revulsives are demanded when the extremities become cool: irritating enemata when the peristaltic movements are sluggish. The illustrious Dr. Pym declares that in no disease are glysters more valuable than in yellow fever,—and this should be an axiom. The tranquillity observed at the commencement in the digestive system is deceptive: in the flaccid belly, and without observable alteration, is included the arsenal of death: the matters contained in these quiet intestines rapidly augment the material of decomposition to be absorbed; in these intestines are diffused the elements of urea when the kidneys no longer eliminate them: I urge that these combustibles be removed in time, and the vicarious routes for the elimination of *urea* and other effete matters be opened. For this object it is necessary to stimulate the urinary secretions as well as the intestinal secretions and excretions, because through these functions and diaphoresis the *materia prima* (which later would give rise to black vomit) are almost always eliminated.

"Such, in my opinion, are the great indications to be fulfilled: there are also special indications to be met during the disease and convalescence.

"The following is a synopsis of the means employed in the Maritime Hospital of Santa Isabel, to fulfil the above therapeutic indications.

"*First period*,—Oleum ricini, magnesiæ sulphas, sodæ sulphas, soluble tartar, citras magnesiæ, calomel, senna, oleum tiglii, diaphoretic infusions, acetate of ammonia, tincture of aconite, of digitalis, belladonda, nitre, lauro-cerasus, Labaraque's solution, opium, clysters of Persicaria (i. e. *polygonum anti-hæmorrhoidale*, native of Brazil) with sulphate of soda or table salt and castor oil, sinapisms, *sinapised* baths, dry frictions or with tincture of camphor, dry or cut cups, leeches to the mastoid regions (seldom,) emollient baths containing tinct. of camphor, sulphuric, citric, or muriatic lemonades, barley water, infusion of flaxseed, tartar emetic, sulphate of quinine, and (when of an intermittent character) baths of Pâo Pereira."

*Geissospermum vellosii*, Dr. Francisco Freire Allemâo. A tree of Brazil which attains to a hundred feet in height, the bark of which is employed by the Indians and others in the interior in the case of intermittent fever, has been recently introduced into the Materia Medica of Brazil, and various physicians of Rio de Janeiro recognise it to be a valuable tonic. A pharmaceutist of that city, Ezequiel Correa dos Santos, obtained from the bark in 1838 an alkaloid, named *Pereirina*, which is supposed to contain its active principle. It is insoluble in water, but soluble in ether, alcohol and acids. It is exhibited in decoction, one ounce of the bark to twelve of liquid internally, and as a bath, one pound of the bark to a sufficient quantity of water.*

*Second period.*—Continued use of mild laxatives, especially tartrate of potassa, citrate of magnesia, dry or scarified cups, leeches on the mastoid apophyses, and to the anus (rarely) nitrate of potassa, cherry laurel water, tincture of aconite, belladonna, opium, lemonades made with sulphuric, citric, nitric or muriatic acid, flaxseed cataplasms with laudanum, emollient baths, hot baths of Pâo Pereira, sudorific infusions, sinapised pediluvia, large volant sinapisms, enemata of Labaraque's solution, cold drinks, camphor, vesicatory to the epigastrium, and sometimes to the lower extremities, fomentation to the belly, unguents of belladonna, mercury, laudanum, cicuta, &c.

Sulphate of quinine, serpentaria, decoction of bark with citric acid, dry frictions or with tincture of valerian, or bark, camphor,

* Formulario ou Guia Medica por Pedro Luiz Napoleâo Chernoviz U. S. D., &c. Rio de Janeiro 1852.

creosote, sinapisms to the trunk and extremities, enemata of Persicaria (Polygonum antihæmorrhoidale) bark, camphor; cataplasms of aromatics, hot bottles passed over the whole surface.

Infusions orange leaves, orange flower water, paregoric, and æther.

In black vomit, blister to the epigastrium, Labaraque's solution very much diluted, lemon juice, a weak solution of ergotine, cold lemonades, sometimes cherry laurel water.

Hæmorrhages were combatted with ergotina, vinegar, Jequitiba, (an astringent bark of Brazil, *Pixydaria macroparpa*, Schott.) Monesia (*Chrysoyhyllum buranhem*, Riedel).

*Third period.*—The remedies used in the third period are the same as those for the second, employed with more or less energy, according to circumstances.

Dr. Lallemant states that by an approximative calculation there were 100,000 cases, and 10,000 deaths.

Dr. Chernoviz states that from the invasion of the epidemic, that is, from the 1st of January 1850, until the 31st of August, the deaths from yellow fever in Rio de Janeiro numbered 3,827; from all other diseases 4,993, or a total of 8,820, including slaves and free.

In the year 1849, when there was no epidemic, the number of deaths was 7,905, which would give for the first eight months 5,270. By comparison it is seen that in the first eight months of 1850, the mortality was 3,550 greater than for the same period in 1849.

The epidemic began to decline about the end of May, and seems to have disappeared early in September. But recurred again in the succeeding years. The following tables, deduced from those given by Dr. Candido, exhibit the mortality from yellow fever, and from other causes in Rio de Janeiro, in the years 1851, 1852, 1853, and part of 1854; and that this disease has prevailed with more or less severity during more than four years.

*Mortality in the city of Rio de Janeiro.*

| | 1851 | | | 1852 | | | 1853 | | | 1854 | | |
|---|---|---|---|---|---|---|---|---|---|---|---|---|
| | Different Diseases. | Yellow Fever. | Total. | Different Diseases. | Yellow Fever | Total. | Different Diseases. | Yellow Fever. | Total. | Different Diseases. | Yellow Fever. | Total. |
| January | 628 | 14 | 642 | 659 | 243 | 702 | 796 | 150 | 946 | 742 | 2 | 744 |
| February | 794 | 37 | 831 | 661 | 70 | 731 | 705 | 176 | 881 | 595 | 1 | 596 |
| March | 911 | 60 | 971 | 777 | 303 | 1,080 | 733 | 142 | 875 | 635 | 0 | 635 |
| April | 889 | 165 | 1054 | 683 | 403 | 1,086 | 621 | 153 | 774 | 585 | 1 | 586 |
| May | 733 | 98 | 831 | 624 | 325 | 949 | 651 | 82 | 733 | | | |
| June | 556 | 28 | 584 | 683 | 189 | 872 | 611 | 73 | 684 | | | |
| July | 550 | 9 | 559 | 627 | 93 | 720 | 565 | 26 | 591 | | | |
| August | 572 | 7 | 579 | 643 | 62 | 705 | 572 | 29 | 601 | | | |
| September | 668 | 4 | 672 | 576 | 62 | 638 | 598 | 7 | 605 | | | |
| October | 661 | 19 | 680 | 673 | 37 | 710 | 598 | 7 | 605 | | | |
| November | 641 | 26 | 667 | 623 | 47 | 670 | 621 | 6 | 627 | | | |
| December | 731 | 8 | 739 | 655 | 109 | 764 | 678 | 2 | 680 | | | |
| Totals | 8,334 | 475 | 8,809 | 7,784 | 1943 | 9,727 | 7,722 | 853 | 8,575 | 2,557 | 4 | 2,551 |

The following table includes the national and foreign sailors, from vessels visiting the port of Rio de Janeiro.

*Summary of Yellow Fever cases treated in the Marine Hospital of Jurujuba, now called Santa Isabel, at Rio de Janeiro.*

| | 1851 Opened Jan 1. and closed July 28. 1851. | | | | 1852 Open from Mar. 5 to Sept. 16 & from Nov. 2. till end of year. | | | | 1853 Open throughout the year. | | | | 1854 Open for time reported. | | | |
|---|---|---|---|---|---|---|---|---|---|---|---|---|---|---|---|---|
| | Admitted. | Cured. | Died. | Per cent. | Admitted. | Cured. | Died. | Per cent. | Admitted. | Cured. | Died. | Per cent. | Admitted. | Cured. | Died. | Per cent. |
| January - | 6 | 2 | 4 | 66 | .. | .. | .. | .. | 132 | 74 | 58 | 43 | 49 | 49 | .. | .. |
| February - - | 45 | 22 | 23 | 51 | .. | .. | .. | .. | 93 | 64 | 29 | 31 | 97 | 94 | 3 | 3 |
| March - - | 96 | 76 | 120 | 66 | 308 | 185 | 123 | 39 | 1[illegible]9 | 136 | 33 | 19 | 101 | 98 | 2 | 2 |
| April - - - | 141 | 74 | 67 | 47 | 187 | 107 | 80 | 42 | 251 | 193 | 58 | 23 | 123 | 90§ | .. | .. |
| May - - - | 70 | 46 | 21 | 34 | 105 | 69 | 33 | 34 | 190 | 164 | 26 | 13 | .. | .. | .. | .. |
| June - - - | 25 | 13 | 12 | 48 | 68 | 50 | 18 | 26 | 182 | 149 | 33 | 18 | | | | |
| July - - - | 1 | .. | 1 | 100 | 38 | 26 | 12 | 31 | 68 | 58 | 10 | 16 | | | | |
| August - - | .. | .. | .. | .. | 8 | 7 | 1 | 12 | 119 | 113 | 6 | 5 | | | | |
| September - | .. | .. | .. | .. | 10 | 8 | 2 | 20 | 99 | 95 | 4 | 4 | | | | |
| October - - | .. | .. | .. | .. | .. | .. | .. | .. | 92 | 92 | .. | .. | | | | |
| November - | .. | .. | .. | .. | 27 | 18 | 9 | 33 | 71 | 70 | 1 | 1 | | | | |
| December - | .. | .. | .. | .. | 79 | 43 | 36 | 45 | 46 | 46 | .. | .. | | | | |
| Totals - - - | 484 | 233 | 251* | 51 | 830 | 513 | 317† | 38 | 1.512 | 1,254 | 258‡ | 17 | | | | |

*In the year 1851, the visit to vessels was not established, so that patients were often admitted in a deplorable condition.

†Between May and August, 1852, the visit to vessels anchoring in the port was established by order of the President of the Board of Health (Junta de Hygiene:) the mortality diminished during this period.

‡The visit to vessels, in the steamer Santa Isabel, began March 4th, 1853; from this date the mortality diminished considerably, as may be perceived in the monthly per centum of this year.

§The total number of patients treated during the four first months of the year was 370, of which 5 died, and 33 being still under treatment with good prospect of cure, gives a mortality of 1 per cent. The steamer Santa Isabel continues her daily visits.

